# Human-Centered Design of Video-Based Health Education: An Iterative, Collaborative, Community-Based Approach

**DOI:** 10.2196/12128

**Published:** 2019-01-30

**Authors:** Maya Adam, Shannon A McMahon, Charles Prober, Till Bärnighausen

**Affiliations:** 1 Stanford Center for Health Education Stanford School of Medicine Stanford University Stanford, CA United States; 2 Institute of Global Health Heidelberg University Heidelberg Germany; 3 Bloomberg School of Public Health Johns Hopkins University Baltimore, MD United States; 4 Harvard T.H. Chan School of Public Health Department of Global Health and Population Harvard University Boston, MA United States; 5 Africa Health Research Institute Wellcome Trust Durban South Africa

**Keywords:** human-centered design, health promotion, health behavior, health knowledge, attitudes, practice, community health workers, telemedicine, eHealth, mHealth

## Abstract

Drawing on 5 years of experience designing, producing, and disseminating video health education programs globally, we outline the process of creating accessible, engaging, and relevant video health education content using a community-based, human-centered design approach. We show that this approach can yield a new generation of interventions, which are better aligned with the needs and contexts of target communities. The participation of target communities and local stakeholders in the content production and design process fosters ownership of the content and increases the likelihood that the resulting intervention will resonate within its intended primary audience and be disseminated broadly. Ease of future adaptation for additional global audiences and modification of the content for multiple dissemination pathways are important early considerations to ensure scalability and long-term impact of the intervention. Recent advances in mobile technology can facilitate the dissemination of accessible, engaging health education at scale, thereby enhancing the potential impact of video-based educational tools.

Accessible and engaging health education is a cornerstone of health behavior change. Especially in low- and middle-income countries, increasing access to effective health education can contribute to improved health outcomes. Prior research has identified several characteristics of effective health education interventions. These include the integration of pictures, narratives, and entertainment-education, in which the health messages that make up the educational content are embedded. However, the effectiveness and long-term impact of health messages ultimately depend on how well the end users can identify with the content that is presented. This identification, in turn, is a function of how well the messages correspond to user needs and wants and how this correspondence is communicated through the design characteristics of the health education intervention.

## Background on Health Education Strategies

Health behavior change, which can be motivated by effective health education, has been called “our greatest hope for reducing the burden of preventable disease and death around the world” [1]. Improving maternal and child health through maternal education is especially important, given that more than 5 million children globally still die before reaching their 5th birthday, mostly from preventable illness [2]. Health education efforts aimed at promoting behaviors like breastfeeding and kangaroo mother care lie at the heart of many successful efforts to reduce child mortality but increasing accessibility and dissemination of such health education remains challenging [3-5].

## Prior Successful Strategies in Health Education

*Narrative approaches* to health education have emerged over the past decade as potentially powerful tools for promoting positive health behavior change [6,7]. As one of the longest-standing modes of human communication and knowledge transfer, Schank and Berman [8] suggest that stories function as cornerstones for learning, providing both the context for understanding and internalizing new information as well as a framework for remembering what we learn. Various theoretical models have been used to characterize the mechanisms by which narrative communication may lead to health behavior change, including the extended Elaboration Likelihood Model (ELM) [9]. In this model, the effectiveness of peripheral processing of the health messaging depends on the viewer’s identification with the characters and their engagement (ie, absorption and transportation) with the storyline [9].

One particularly successful application of the narrative approach to health education can be seen in *entertainment-education* (E-E) [10]. E-E involves the delivery of health messages by embedding them in entertainment media. A substantial body of research suggests that E-E is an effective way of influencing beliefs, attitudes, and behaviors [9-13]. This approach may be even more effective in populations with lower motivation and ability to cognitively evaluate the health messages being delivered [10]. E-E media is particularly impactful when the following characteristics are incorporated: (1) an appealing storyline, (2) high-quality production, (3) unobtrusive persuasive messages, and (4) high potential for involvement with the characters.

Even the use of *simple pictures*, without an accompanying narrative storyline, has long been recognized as an effective way of enhancing attention to, and recall of, health education [14]. A significant body of prior research suggests that appropriate implementation of pictures can positively influence patient responses to health instructions and improve their health behaviors [14-16]. *Emerging technology* including mobile and online education platforms [3,5,17] present new opportunities for the dissemination of *video-based health education*. In recent years, researchers have begun to document the growing potential for online learning platforms, mobile phones, and tablets to fulfill important functions in the dissemination of open-access health education [17-20]. The increasing availability of mobile technology in low- and middle-income countries (LMIC) foretells promising new avenues for the delivery and scaling of mobile health education to communities who need it urgently [3,5,17,21,22].

## Human-Centered Design of Global Health Education

Since 2013, a team of health educators at the Stanford University School of Medicine and the Stanford Center for Health Education has been grappling with a challenge: how do we create optimally effective and engaging video-based health education tools and deliver them at scale by adapting them across a broad spectrum of global learners? Over a 5-year period, our target audiences have ranged from medical students and practicing physicians to community health workers in LMIC and the general public. Dissemination pathways for our content include university learning management systems, open online learning platforms, mobile apps, government clinics, and nongovernmental health organizations.

The goal of creating content that is accessible, engaging, and relevant to the needs, wants, and experiences of the end user has been central to our learning trajectory. This has ultimately led us to apply a human-centered design (HCD) approach to the creation of our content and programs. HCD of health education revolves around several principles described in the literature, including empathy with the target communities, a process of rapid prototyping, feedback gathering and responsive iteration [23,24], as well as a tolerance for ambiguity and failure throughout the design process [25-27]. HCD involves integrating local characters, visuals, and narratives into accessible content, designed to deliver health messages that are collaboratively defined and created. A key principle of HCD is the idea that successful solutions should be created with the needs and wants of the end user in mind. Aligning with this principle, our content creation process has developed over time to rely increasingly on multiple cycles of feedback from community members in our target audience (our end users) and rapid iteration in response to that feedback. Especially in under-resourced and low-literacy settings, we have found that taking a community-based approach, by involving the target audience in the content creation process, is more likely to yield health education programs that are truly rooted in the contexts of the communities they intend to serve. Furthermore, through the creation of video-based teaching tools featuring narratives and visual elements that resonate with end users, we believe we can optimally engage our target audiences, empowering them to improve their own health behaviors and ultimately, their own health outcomes. The positive global response to our work contributed to the founding of the Stanford Center for Health Education in 2017 and its nonprofit global health education arm, the Digital Medical Education International Collaborative [28]. Overall, 8 video-based health and medical education initiatives, developed by our team over a 5-year period, generated the guidelines presented in this paper. These formative projects are described in Table 1 and presented along a development timeline map [29]. Multimedia Appendices 1-11 are referenced within this table and contain relevant video samples.

**Table 1:** Health and medical education initiatives (2013-2018).

Content subject matter	Technology, skill requirements, and scope	Audiences with delivery platforms, reach, and sample content	Collaborators, design approach, and feedback summary
Basic physiology and child health (2013)	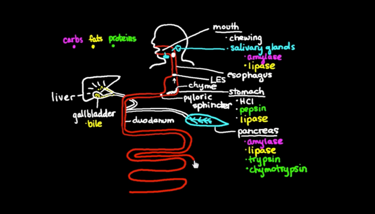 Basic digestion: sample physiology teaching video created in 2013 (Multimedia Appendix 1). Overall, 14 videos, 9 to 12 min each; amateur digital illustrations on black background; captured using screen-capture software (Camtasia); simultaneous audio capture via Rode microphone	Undergraduate students in a flipped-classroom Stanford Human Biology 121 course (content accessed by approximately 400 Stanford students via unlisted YouTube playlist [30]); learners in the general public (via The Khan Academy website [31] and The Khan Academy Medicine YouTube channel [32]), number of content views range from 43,524 (asthma [33])to 893,899 (basic metabolism [32])	Exploratory content; created by a single faculty member; modeled on Khan Academy teaching style; employed a purely didactic teaching approach; faculty content creator did not use a human-centered design approach; content was relatively cost-effective to produce: no professional illustrator was needed because basic editing was done by faculty content creator; students responded favorably to gradually unfolding visual elements, liked having the ability to pause, rewind, or watch at 2x speed if needed; black background wasted too much ink for students who wished to print out end screen as a study tool (feedback: public comments can be viewed here [32])
Infectious diseases (microbiology and immunology, 2014)	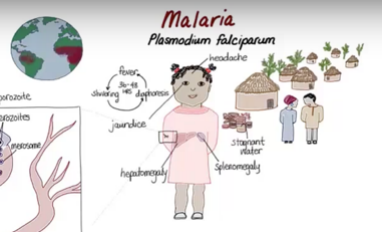 Malaria teaching video created in 2014 for Stanford Medicine's infectious disease course (Multimedia Appendix 2). Overall, 36 videos, 8 to 10 min each; amateur digital illustrations on white background to facilitate printing of end screens; captured using Camtasia; asynchronous, scripted audio capture (via Rode microphone); subsequent synchronizing of audio and visual components in postproduction	International medical students as part of required microbiology/immunology course offerings (via university learning management systems and Stanford Medicine YouTube Playlist [34], 3000+ playlist views); international health sciences students (via Stories of Infection [35] open online course on Coursera, current enrollment: 17,116)	Content created in collaboration with faculty experts and medical students at Stanford University, University of California San Francisco; Duke University, University of Washington; and University of Michigan; formative feedback from medical students collected via focus groups throughout the course development period; medical student advisors attended weekly meetings, participated in script-writing and storyboarding process; medical student content users reported that the patient-centered narrative approach, interwoven with didactic elements, provided a useful framework for remembering the material and preferred a white background for easier printing of end screen as a study tool; (feedback from international Coursera learners: 99% positive, based on 4904 ratings)
Child nutrition and cooking (2014)	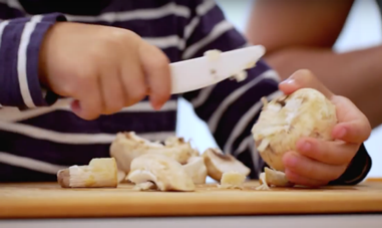 Stanford's Child Nutrition and Cooking course was created in 2014 (Multimedia Appendix 3). Overall, 46 videos, 4 to 9 min each; amateur digital illustrations used in didactic videos + multimedia entertainment-education (E-E) cooking demonstrations; on-camera segments shot and edited by professional videographer	Undergraduate students in a flipped-classroom Stanford Human Biology 81 Q (content accessed via YouTube playlist [36]); international parents and caregivers (via the Stanford Child Nutrition and Cooking [37] open online course on Coursera), current enrollment: 315,834.	Stanford School of Medicine Faculty Nutrition Experts collaborated with parents and teachers at Stanford’s Bing Nursery School; children and local celebrity chefs also featured to stimulate and empower parents to apply principles presented in nutrition education videos; parents responded favorably to positive role modeling, inclusion of children and celebrity chefs, and the connection made between theory and practice of child nutrition; adults without children requested a similar course on adult nutrition topics (feedback from international Coursera learners: 99% positive, based on 12,918 ratings)
Community maternal and child health (2015-18)	14 videos, 3 to 4 min each; digital illustrations using reveal animation (illustrations masked, then revealed to mimic live drawing, using Premier Pro software); illustration and editing support required; content adapted for: (1) South Africa in isiXhosa 2015 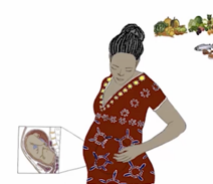 Xhosa nutrition in pregnancy video created for South African maternal child health promotion in 2015 (Multimedia Appendix 4) (2) India in Hindi 2017 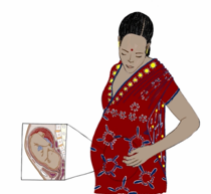 Hindi nutrition in pregnancy video adapted for Indian maternal child health promotion in 2017 (Multimedia Appendix 5) (3) Burkina Faso in Dioula 2018 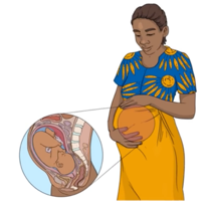	Community health workers (CHWs) and their clients in (1) South Africa 2015: content [38] delivered via CHWs at the Philani Maternal Child Health and Nutrition Trust (Philani); India 2017: content [39] delivered via tablets and projector at Antara Foundation and Vivikenanda Tribal Hospital; Burkina Faso (content [40] pilot, 2018); app for Android devices created in 2015 [41]	Stanford School of Medicine Health Educators collaborated with CHWs and supervisors at the Philani Maternal Child Health and Nutrition Trust in Khayelitsha, South Africa; didactic and story-based maternal-child health education videos; illustrated by local artists and students; professional voiceover service used for the early videos: relatively expensive, language delivery did not consistently resonate with end users; 2016: began using the voices of CHWs and supervisors; content area priorities and scripts collaboratively developed and translated by Philani staff; Philani CHW end users responded favorably in a 2016 qualitative feasibility study [42]; feedback pending from adaptations created for India and Burkina Faso
Food and health for adults (2016)	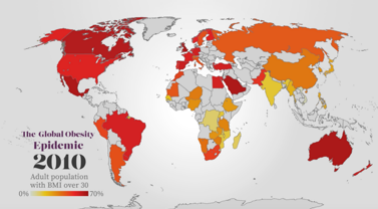 Stanford's Introduction to Food and Health course, created in 2016 (Multimedia Appendix 6). Overall, 28 videos, 4 to 6 min each; multimedia, E-E approach; video footage and animations; 10 on-camera cooking demonstrations (massive open online version only); film-maker and professional animator engaged to support course creation	Stanford medical students as part of their nutrition unit (course content accessed via Stanford’s university learning management system); practicing physicians via continuing medical education (CME) course (course content [43] accessed via Stanford Online CME platform); international learners (via YouTube playlist [44] and the Stanford Food and Health [45] open online course on Coursera, current enrollment: 183,229)	Stanford School of Medicine Faculty Nutrition Experts collaborated with a celebrity food journalist, local animators, and a food/lifestyle channel; video series integrated animation and multimedia, E-E; Course piloted by Stanford medical students in required nutrition block; students and international learners responded favorably to integration of celebrity food journalist’s perspective and E-E approach; (feedback from international Coursera learners: 98% positive, based on 21,087 ratings)
Gender identity and children’s health (2017)	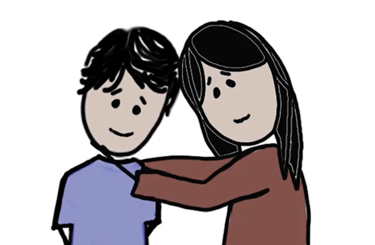 Health Across the Gender Spectrum (massive open online course) 2017 (Multimedia Appendix 7). Overall, 18 videos, 2 to 6 min each; amateur digitally illustrated videos; narrative approach interspersed with on-camera interviews; videographer and editor needed for live interviews	International learners (via Health Across the Gender Spectrum [46], open online course on Coursera, current enrollment: 12,668) and via YouTube playlist [47]; physicians via Stanford online CME course (pending release of in Oct 2018)	Stanford faculty at the School of Medicine collaborated with local stakeholders: Gender Spectrum, Planned Parenthood, the Stanford University Gender Clinic, Vaden Student Health Center; authentic narratives of transgender children, their parents, physicians, teachers, and 2 acclaimed transgender Stanford academics used to enhance engagement in the course content; interviewees (featured in the videos as animated line drawings, to protect their identities) played a formative role, through Rapid Iterative Testing and Evaluation [23,24], by sharing their stories and participating in the design of their illustrated characters; international learners expressed strong emotional responses to the personal narratives; (feedback from international Coursera learners: 98% positive, based on 1,680 ratings)
Breastfeeding promotion (2018)	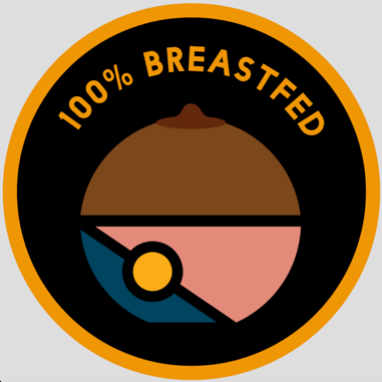 100% Breastfed series launched in South Africa in 2018 (Multimedia Appendix 8). Overall, 4 parallel versions x10 videos, 2 to 4 min each; multimedia (live footage and illustrations); interviews with celebrity and community mothers; videographer, editor, professional illustrator, and logo designer involved	The general public (via Stanford’s Short Course on Breastfeeding [48], an open online course on Coursera, current enrollment: 12,187) and via YouTube playlist [49]; South African mothers, caregivers, and health workers in multiple languages (via open-access, dedicated website [50] for this initiative)	Stanford Center for Health Education established its international education outreach arm, Digital Medical Education International Collaborative (Digital MEdIC) South Africa, with a local team based in Cape Town, led by the first author; video series created in collaboration with United Nations International Children's Emergency Fund; the Western Cape Department of Health; the National Department of Health in South Africa; as well as the University of Cape Town, Stellenbosch University and the Philani Maternal Child Health and Nutrition Trust; series features celebrities and community mothers, who participated in content creation by reacting to early drafts via WhatsApp; Philani CHWs responded positively to the inclusion of celebrities, multimedia approach, and branding of the course as well as its delivery using tablet devices, explored during “bodystorming” [23,51] (physical brainstorming sessions) described below. (Feedback from international Coursera learners: 99% positive, based on 1035 ratings)
National and regional government health education programs (completion in 2019)	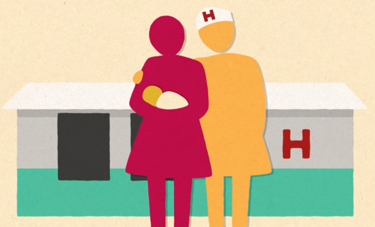 Road to Health series content style: Narrative, icon style, maternal-child health library (75 videos planned, 2 to 3 min each); currently in production (6 videos completed); illustrator-animator and editor involved	The general public with a focus on mothers and caregivers, (dissemination via National Department of Health programs); see samples below: (1) Road to Health Trailer [52]; created for the South African Dept. of Health in 2018 (Multimedia Appendix 9); (2) Breastfeeding video [53]; created for the South African National Dept. of Health in 2018 (Multimedia Appendix 10); (3) Kangaroo Mother Care video [54] created for the South African National Dept. of Health in 2018 (Multimedia Appendix 11)	Digital MEdIC South Africa invited to collaborate with the Western Cape Department of Health, the National Department of Health, the DG Murray Trust, and Ilifa Labantwana to create content in support of South Africa’s Road to Health Book [55]; initiative reaches every new mother who delivers in a health care facility; icon characters designed through a formative process involving Rapid Iterative Testing and Evaluation [23,24], to resonate across ethnic and socioeconomic demographics; story-based, community-narrated approach to the accompanying audio designed to maximize authenticity and identification with the characters; content will be disseminated through the National Department of Health programs

## Adaptability Across Learner Populations, Platforms, and Time

An additional challenge when creating health education content for dissemination at scale is the need to easily adapt that content across different learner populations, different platforms, and across time. Adaptability across different learner populations, including language groups as well as cultural and socioeconomic contexts, is essential for broad scaling of health education content, both within countries and internationally. In community health education, this can mean designing characters that would resonate across ethnic and socioeconomic groups within a given country. In medical education, this can mean including both units of measurement or presenting a symptom generically as “an elevated body temperature” rather than “a fever of 102 degrees Fahrenheit,” which limits the scalability of the content internationally. Easy adaptation facilitates the reach and therefore the impact of each video asset created. This also greatly contributes to the cost-effectiveness of such video-based health education.

The need to easily adapt content for existing and emerging platforms and delivery pathways also requires careful consideration. Especially given the current global trend toward increasing penetration of smartphones and other mobile technology [56], video-based health education should ideally be optimized for mobile dissemination in the future, even if it is delivered in the shorter term using older platforms, such as university learning management systems, DVDs, offline tablets, or in-clinic television screens. As data costs decrease and the number of smartphones increase around the world, effective content needs to remain flexible and easily adaptable for emerging mobile dissemination pathways. This can include zooming in on diagrams or detailed images, such that the main teaching points can easily be appreciated even on smaller screens. Decisions about the nature of the visual media included in health education videos will also have enormous implications for file size, with the inclusion of video footage and high-resolution artwork resulting in much larger file sizes than more simple visual styles. Given the relatively high cost of data in some LMIC, file size can have significant implications in terms of adaptability for mobile push messaging.

Finally, because of the significant investment of cost and time in producing high-quality video content, this medium is best for disseminating health and medical educational content that will likely remain “evergreen” for several years. This implies selection of evidence-based concepts that are both foundational and unlikely to change considerably in the short term. For example, a health education video about the benefits of breastfeeding would likely have greater longevity than a video summarizing the differences in breastfeeding rates by country, as these are much more likely to change from year to year. Even subtle language choices can facilitate the longevity of video-based educational content. For example, “Until 2016, the World Health Organization categorically advised HIV positive mothers against mixed-feeding, regardless of their ARV [antiretroviral] treatment regimens” facilitates longevity better than “Until 2 years ago, the World Health Organization categorically advised HIV positive mothers against mixed-feeding, regardless of their ARV [antiretroviral] treatment regimens.”

## South Africa as a Case Study

In South Africa (SA), a country with 11 national languages and a population that is ethnically, culturally, and socioeconomically diverse, a community-based, HCD approach has been instrumental in addressing the challenges of engagement and adaptability. The 2 projects described here illustrate the application of such an approach.

### The 100% Breastfed Initiative

The 100% Breastfed Initiative, launched in March 2018, consisted of a series of 10 breastfeeding educational videos targeted primarily toward SA mothers. During the formative, early discussions with local health workers and community members, it was suggested that the educational series should include on-camera interviews with celebrity mothers who would serve as role models and representatives of SA’s ethnic diversity. Feedback from 2014 and 2016 massive open online courses (Table 1) suggested that the inclusion of the celebrity perspective seemed to enhance engagement within the general public. Overall, 3 SA celebrity mothers were identified, interviewed, and included in early draft videos.

Community members and local clinics viewed the content and suggested adding the perspectives of community mothers to emphasize that many of the challenges faced by new mothers in SA transcend these demographics. This suggestion was accepted and implemented in the next iteration of the video series. Freehand, digital illustrations, drawn by a local SA artist, and branding (including a logo, see Table 1) were later also integrated in response to input from a large SA women’s media publishing house. The illustrations were intended to underscore the cross-cultural nature of the health messages, by reflecting South Africa’s ethnic diversity. The logo and branding were intended to facilitate national dissemination of the campaign.

A total of 4 versions of the course were created in parallel for different audiences between July 2017 and March 2018 (English for SA mothers, isiXhosa for SA mothers, English for SA health workers, and an international version, launched on Coursera). The scripts for each teaching video were created in real-time collaboration with 11 local stakeholders using Google Drive to manage version control. For each video, an audio-visual script [57] showing 3 parallel versions would be finalized before production would begin. Version_2 (advanced) was later converted into 2 versions, one narrated by twin SA medical doctors, Vela and Phinda Njisane, for SA health workers and the fourth version narrated by a Canadian health educator, for a massive open online audience.

As early drafts of the videos were completed, these were shared with local community advisors using WhatsApp, a communication app that supports the transfer of video files. This feedback led to several iterations of the images, characters, and content, a process that continued throughout the production period. WhatsApp was a significant production asset, useful in soliciting feedback from community members who did not regularly use email and did not feel comfortable editing scripts using Google Drive.

Final content prototypes were loaded onto android tablets to be used by Community Health Workers in physical brainstorming sessions, described as “bodystorming” [23,51] in prior HCD literature. During these sessions, community health workers engaged in role-play that involved testing the intervention in mock-counseling sessions, observed by members of the production team.

The final video series was launched in March 2018 and has since also been translated and dubbed in Afrikaans. Over the next 12 months, the content will be translated into several other national languages.

A cluster randomized controlled trial involving 1008 pregnant women was launched in September 2018 to study the effects of the 100% Breastfed series on breastfeeding rates in the study participants.

Although the videos have been well received to date (see Table 1), we realized that this approach had some limitations. First, the file size of videos containing live footage is considerably larger than those containing simple illustrations or animations. This makes dissemination via mobile push messaging to smartphones prohibitive at present, due to the relatively high cost of data in South Africa. Second, adapting the content for use in other African countries would require identifying and interviewing local mothers in each country, then re-editing the series to give it a local flavor. Although the freehand, digital illustrations are somewhat easier to adapt, they would also require redrawing for each target country to accurately represent different styles of dress, local facial features, local foods, etc. We encountered similar issues when adapting the 2015 Community Maternal and Child Health course (originally created for an under-resourced urban South African population) for audiences in India (2017) and Burkina Faso (2018). Although live footage and realistic depictions of characters can be powerful ways to enhance visual identification with characters, this approach also limits the ease of adaptation of the resulting content, an especially important consideration in countries or geographic regions that are home to multiple diverse ethnic and socioeconomic groups.

### The Road to Health Initiative

The Road to Health Book (RTHB) is a child health initiative of the South African National Department of Health, first launched in 1973 and updated for relaunch in 2018. The goal of the initiative is to provide new mothers across South Africa with education and resources to help them become more engaged in their child’s health, in partnership with their child’s health care providers.

In 2018, we were invited to begin creating video-based content to support the RTHB initiative. The main challenges were (1) to create content that would resonate across SA and (2) to dramatically reduce the file size of the video content because the data charges associated with using the content would be reverse billed to the National Department of Health.

Formative discussions with local SA stakeholders in maternal-child health, as well as feedback from earlier online courses, helped to distill a central goal: to create a visual style that was extremely simple (yielding smaller video file sizes) and universal enough to resonate across ethnic and socioeconomic groups, ideally with the possibility of repurposing the content for a global audience in a cost- and time-effective way.

While retaining visual simplicity, the content needed to elicit enough emotion in the viewer to engage a lay audience who was choosing to watch the content voluntarily (ie, this was not required viewing as part of a formal educational program). Feedback from a 2017 massive open online course (Table 1) suggested that the inclusion of dialogues between family members or individuals involved in close interpersonal relationships was a powerful way to engage learners, even in topic areas where initial motivation to attend to the content was low.

Over the following 4 months, we solicited feedback on different iterations of simple icon-style characters through a process of rapid iterative testing and evaluation described in the HCD literature [23,24]. Each resulting prototype served as the new design direction until consensus was reached on a modified version of the “universal human” icon that is used globally in public places, most commonly to indicate the location of restroom facilities. Table 2 summarizes the prototyping pathway. Visually, these icon-style characters were enhanced through the use of various nonskin tone colors and textures. Their settings were kept similarly universal by relying on simple shapes and lines to represent props in the environment, such as eating surfaces or places to sit.

**Table 2:** Visual development progression.

Visual style development	Visual considerations and feedback
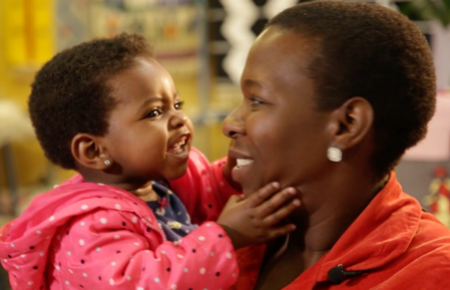	Live footage of interviews with community members and local celebrity mothers used to enhance engagement in the 100% Breastfed Health Education series; changing voiceovers allowed for in-country adaptation for different learner populations but adapting the course for other African countries would require significant resources as on-camera interviews would need to be rerecorded for each new country
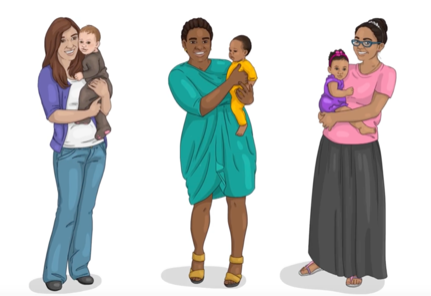	Freehand digital illustrations by local illustrator featured South African (SA) mothers and families; characters designed to represent varying demographics to enhance identification with the content broadly across SA; each teaching video incorporated different characters, underscoring the common experiences and challenges faced by new mothers, related to infant feeding
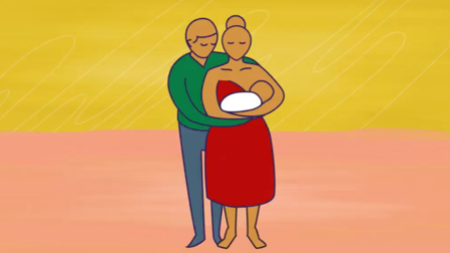	Icon-style concept art (iteration 1) developed to elicit feedback on the icon approach; visual style not favorably received by community members and local stakeholders: (1) Characters appeared to belong to 1 ethnic group due to skin color and hair (with limited adaptability for non-Western audiences) and (2) clothing styles were also thought to be unacceptable for many non-Western audiences
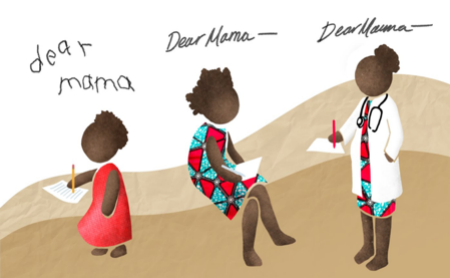	New icon-style concept art (iteration 2) developed; visual style favorably received by community members and local stakeholders, with some limitations: (1) textures and simplicity of images found to be acceptable, with neutral background and concept of *universal surfaces* found to be preferable to specific props like table/chairs and (2) clothing style and visible ethnic cues like skin color and hair could limit ease of adaptability for other cultures
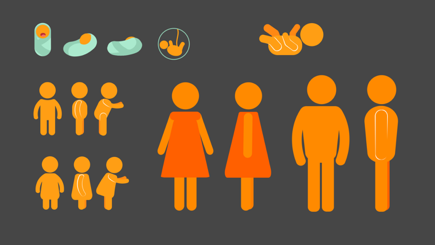	New icon-style concept art (iteration 3) developed; visual style somewhat favorably received by community members and local stakeholders, with some limitations: (1) simple icon characters thought to be more easily generalizable across different cultures and demographics and (2) early feedback suggested that images may be too juvenile (“cartoony”) for adult learners and might too closely resemble emojis
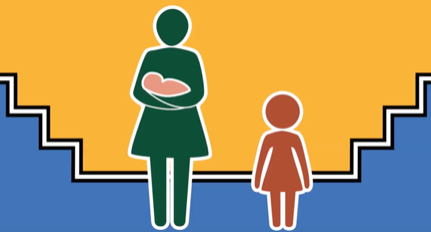	New icon-style concept art (iteration 4) developed; prototype resonated with national government and nongovernmental organizations as well as community advisors: (1) neutral background incorporating *universal surfaces* from iteration 2 thought to be appropriate for SA due to subtle Ndebele (African painting style) through a double black line; (2) lack of visible ethnic or socioeconomic identifiers found to be an asset; (3) additional benefit: colors could be adapted as needed to match various health campaigns
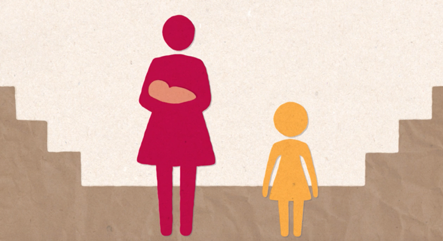	New icon-style concept art (iteration 5) developed; prototype approved for use in the Road to Health Book initiative by the National Department of Health and resonated with community advisors: (1) prototype incorporates *universal surfaces* neutral background and character design free of ethnic and socioeconomic identifiers, (2) color scheme easily altered as needed, (3) drop-shadowing makes texture icons appear somewhat three-dimensional (without adding to production costs) when paired with color, animation, and voiceovers

Localization of this new video content style relies almost entirely on the audio components, which feature the voices and narratives of the target audience as well as a soundtrack. By recruiting the end users of the content to help shape and deliver the voiceovers, these videos acquired an authentic, community-based quality, according to early, informal feedback from SA community members.

The next steps will include conducting formal focus groups across different language groups as part of the National Department of Health’s evaluation of the RTHB initiative.

The main advantage, from a production perspective, of employing a simple visual style with reliance on the voiceover and narrative for localization, is that this approach is easily adaptable, by changing the audio components only. Furthermore, involving the end user (or target community) in the voiceover recordings and intervention development can serve to empower these communities and facilitate ownership of both the educational content and the health behaviors they aim to promote.

## Theoretical Framework

This work rests upon the theoretical underpinnings of the ELM [58], which suggests that there are 2 contributing pathways to achieve the changes in attitude that predict a desired behavioral outcome. The first *central route* is influenced by the learners’ motivation and ability to cognitively process the information presented. This can be influenced by factors such as the length of the content and the degree to which the language used is accessible to the learner. The second *peripheral route* relies on cues embedded in the method of information delivery that contribute to its relative acceptability to the learner. Positive, peripheral cues, such as the learners’ subjective evaluation of the narrator or the learners’ emotional involvement in the content, lead to peripheral attitude changes, which, although less enduring than central attitude changes, can positively influence the learners’ motivation to process the messaging via the central route [58].

## Production Guidelines

In this section, we outline the steps we have followed when creating video-based health education content, using HCD. These production guidelines help to standardize the video production process across projects and teams as well as supporting quality control and time management. The guidelines were developed collaboratively, over 12 months, by content creation leads and creative team members in the United States and South Africa. The guidelines are based on prior research on the effects of different video production strategies on learner engagement [59]. To some extent, the desired style of content being produced will define the video production workflow [60], as well as the production team skill sets and the budget needed to support such work. For example, creation of the storyboarding and animatic steps, described below, would primarily be text-based descriptions of anticipated footage for a video integrating mostly camera footage, whereas the storyboard and animatics would primarily consist of draft illustrations for an animated video. In Table 3, we describe our workflow as well as provide examples and strategies for optimizing each phase. Multimedia Appendices 11-14 are referenced within this table and contain relevant video samples.

## Takeaways

Applying a community-based, HCD approach during the collaborative development of scalable video-based health education content can yield significant benefits including:

- Target communities are empowered as the strengths and resources within them are identified, harnessed, and showcased. Recognizing the human resources already available within a community, by involving them in the production of their own health education content, serves to validate, educate, and enable that community. Empowered community members can promote health within their peer groups and can advocate for increased access to resources like health and social services on behalf of their communities. An empowered community recognizes and prioritizes health education and the behaviors associated with improved outcomes. They also become valuable partners in the dissemination of their own health education content.
- This approach fosters improved health awareness, increases identification of the target communities with the content, and creates a sense of ownership among community members who participated in the production of that content. General health awareness improves as a result of peer-to-peer education (word-of-mouth) between those involved in the content creation and their fellow community members. A sense of ownership results from the fact that time and energy have been invested by the community in the content creation process. Identification is enhanced by the fact that content better reflects the context, needs, and wants of the target community. An increased sense of awareness, ownership, and identification with the content decreases resistance to health messages (also called counterarguing) as the messages are perceived as coming from sources that have been internally validated rather than being foreign and external to the community.
- Localization through the incorporation of local narratives and simple visuals yields content that can be adapted for other audiences in a cost- and time-efficient manner. Incorporating local stories and the voices of community members results in content that aligns desired health messages with local beliefs, perspectives, and language used to communicate around these issues. As such, content relies more on audio than visual components for its cultural resonance and can be easily adapted for other audiences—often simply by changing the narrative voiceover. Easier adaptation, in turn, facilitates scaling of health education to other regions. Furthermore, simplifying visual components reduces the financial burden associated with transferring large data files.

**Table 3:** Production workflow.

Main objectives	Description	Example
Instructional design phase	Define target audience for final video (1) identify general intent/purpose of the video for each audience, (2) specify learning objectives, (3) decide on the target length of video, (4) define visual style of video, (5) define pedagogic approach (ie, narrative, didactic, or hybrid), (6) create a design document [61]	The Grow Great design document [62] was created in preparation for a collaboratively developed growth stunting reduction campaign. Video content developed in collaboration with the DG Murray Trust, South Africa. 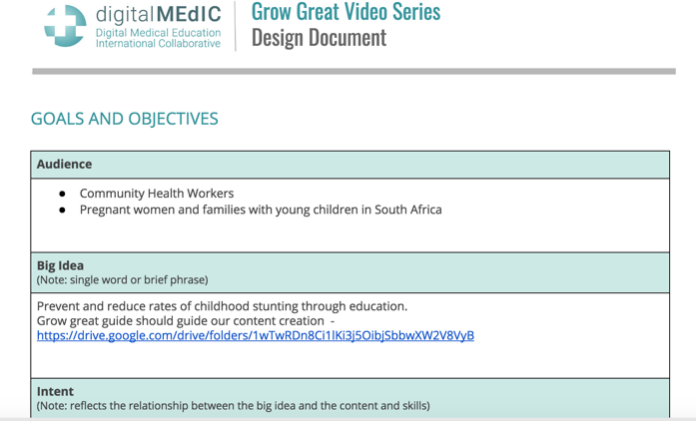 Sample template header for design document
Drafting of an audio-visual script	Consider how the learner will be guided through the learning objectives, including which background information may need to be provided; draft core dialogue if the video will be “scripted” or record interviews if teaching scripts will be derived from recorded interviews.	Audio-visual script [63] for Grow Great Video 1. Prescripted teaching video [54]; (Multimedia Appendix 11); teaching video derived from a recorded interview [64] (Multimedia Appendix 12) 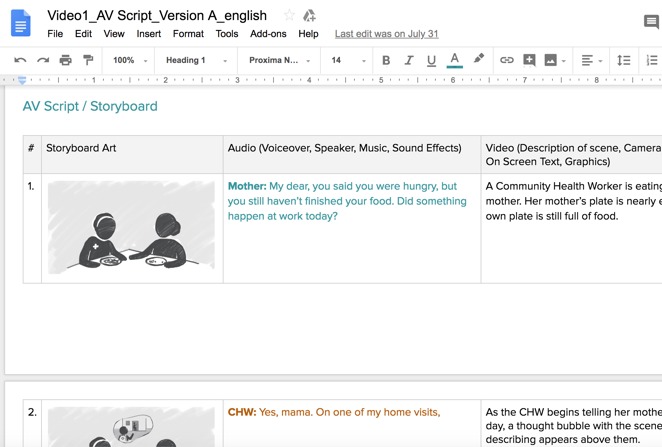 Audio-visual script with storyboarding
Storyboarding	Plan accompanying visuals for each frame (each sentence or main point of the video); generate rough sketches of illustrations to be used; obtain video footage or other images if relevant	Audio-visual script [63] (with storyboarding) for Grow Great Video 1
Obtaining feedback from stakeholders and end users	Share A/V script with relevant local stakeholders and selected creative advisors within the target community. (Feedback can be collected via Google Drive, videoconference with screen sharing or Word documents by email with “track changes” to manage version control); mobile communication tools that support video transfers (such as WhatsApp) are valuable for soliciting feedback from target community members who do not regularly use email.	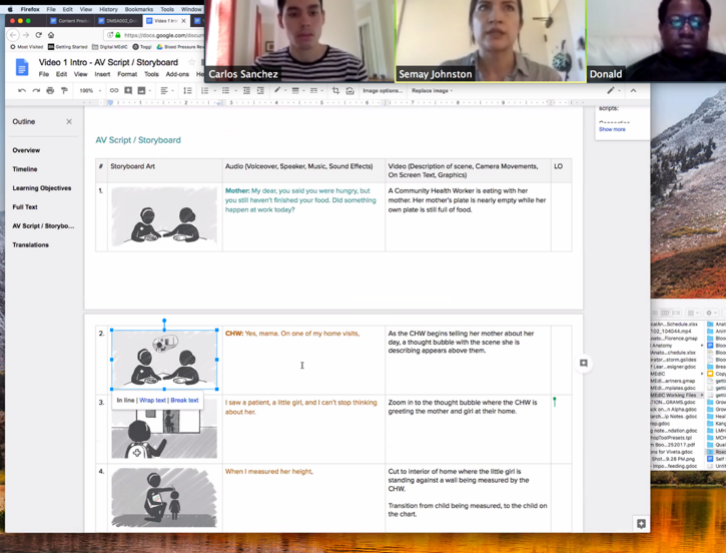 Feedback session in USA and South Africa via Zoom videoconference with screen sharing
Audio recording	Identify community members from within the target community to record the voiceover for the video. (This process may involve *auditioning* interested community members, with external feedback from other end users informing the selection of finalists); consider that the quality of the audio recording can vary depending on the available recording equipment and location. If resources are limited, a voice-recording app on a smartphone can be used to record audio scripts inside a closed car, parked in a quiet area. Smaller, insulated spaces can be adapted to yield clean audio with little or no echo (ie, closets containing clothing or blankets hung on walls to absorb sound waves). If available, a soundproofed recording booth with a high- quality microphone is preferable; community members may need *coaching* to deliver lines in a way that sounds authentic and compelling. (Tips: encouraging community voice performers to stand while recording and use facial expressions, such as smiling and raising eyebrows, can yield a more convincing delivery.) Local celebrities can be recruited as compelling voice talent for public health education interventions.	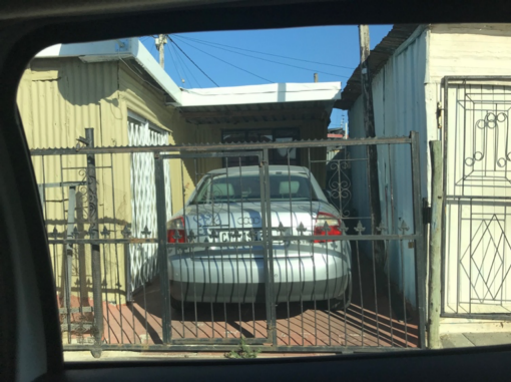 Makeshift “Recording Studio” In Khayelitsha, South Africa 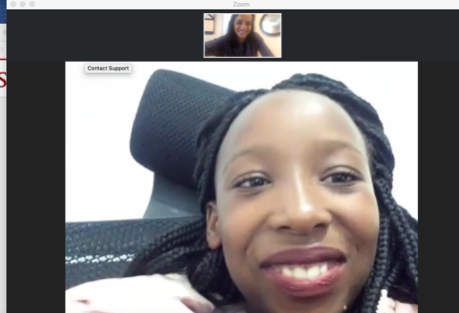 Virtual recording session with community voice talent, Qhawe Nkopane (age 10 years) 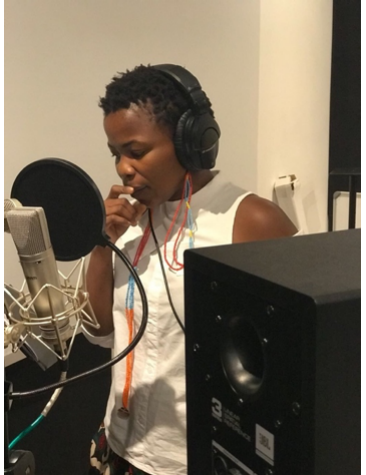 South African celebrity Zolani Mahola (lead singer of the band *Freshlyground*) volunteered to narrate the 100% Breastfed series, Cape Town, 2018
		
Animatics (creating sequences of images or sketches)	Combine still frames of storyboard with recorded audio using editing software such as Camtasia, Premiere Pro, or others; use video collaboration review tools such as Frame.io for internal review and feedback from local stakeholders; use WhatsApp or face-to-face meetings to gather feedback from end users who are more comfortable with these feedback avenues; use *bodystorming* sessions to role-play the use of the intervention in the field by end users.	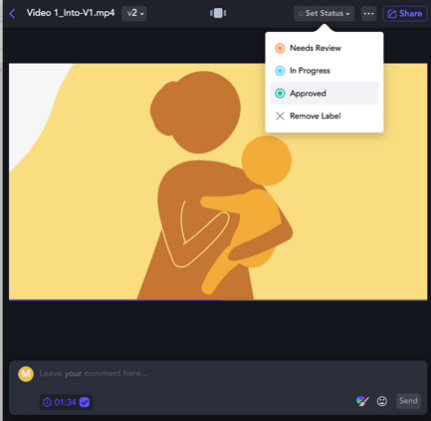 Frame.io [65] is an online video review and collaboration tool. An “animatic” [66] (Multimedia Appendix 13) is a draft video created using rough sketches only. 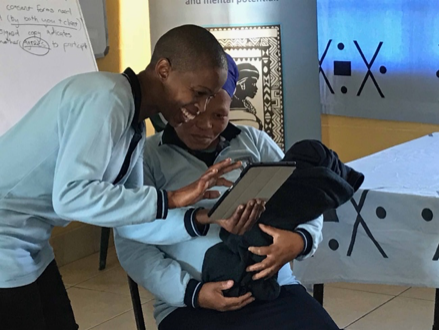 Community health workers engage in role-play exercises to test the use of the intervention for future home visits with new mothers. (Simple props such as the “baby” in this scenario can help with early identification of potential barriers to implementation.)
Art assets	Digital illustration tools such as Photoshop or Adobe Illustrator are useful; if animations will be incorporated, specify the naming conventions for layers and individual elements to be animated, for example: “character_bodypart” (eg, girl_head, girl_body, girl_armL, girl_armR, girl_legL)	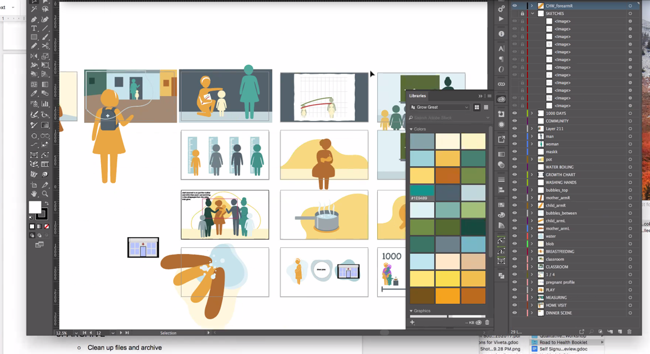 Digital illustration tools such as Photoshop or Adobe Illustrator can be used to create simple images that underscore teaching messages and enhance engagement.
Animation (art assets edited to create the illusion of movement)	If animation used, *After Effects* and/or *Premiere Pro* can be useful software assets; use animatic as a guide layer.	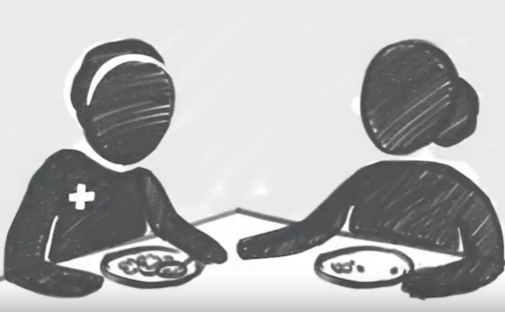 An “animatic” (Multimedia Appendix 13) can be used as a guide layer for animation.
Delivery	Render (export) videos for high definition and mobile viewing; upload to Frame.io and Google Drive for sharing and a final round of feedback.	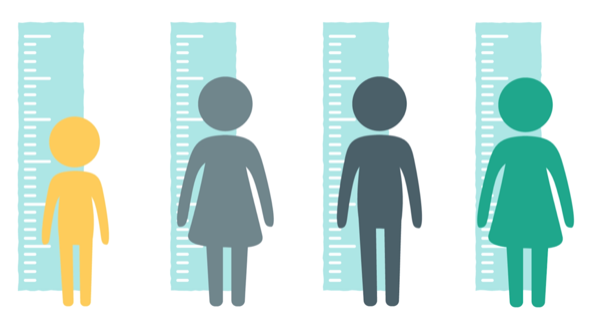 Growth stunting awareness video created with the DG Murray Trust, South Africa, 2018 [67] (Multimedia Appendix 14)
Archiving	Name final videos according to consistently defined conventions (ie, FINAL_ Grow Great_1_July18); file video creation documents and all iterations for future look-back; file art, music, and sound assets in a production library for reuse in future projects.	

Human beings are hardwired to learn through stories and personal narratives. By collaborating with target communities to (1) identify and shape relevant narratives, (2) harness local talent and expertise to convey priority health messages through them, and (3) “digitally package” the resulting content for easy, open-access global scaling, we can marry the oldest and newest forms of teaching in a synergistic and innovative approach to health education.

## Appendix

Multimedia Appendix 1 Basic digestion: sample physiology teaching video created in 2013.

Multimedia Appendix 2 Malaria teaching video created in 2014 for Stanford Medicine's infectious disease course.

Multimedia Appendix 3 Stanford's child nutrition and cooking trailer was created in 2014.

Multimedia Appendix 4 Xhosa nutrition in pregnancy video created for South African maternal child health promotion in 2015.

Multimedia Appendix 5 Hindi nutrition in pregnancy video adapted for Indian maternal child health promotion in 2017.

Multimedia Appendix 6 Stanford's Introduction to Food and Health trailer, created in 2016.

Multimedia Appendix 7 Trailer for health across the gender spectrum (massive open online course) 2017.

Multimedia Appendix 8 100% Breastfed trailer, launched in South Africa in 2018.

Multimedia Appendix 9 Road to health trailer created for the South African Department of Health in 2018.

Multimedia Appendix 10 Breastfeeding video created for the South African National Department of Health in 2018.

Multimedia Appendix 11 Kangaroo mother care video created for the South African National Department of Health in 2018.

Multimedia Appendix 12 Teaching video (derived from a recorded interview) created for open online course: Health Across the Gender Spectrum.

Multimedia Appendix 13 Sample animatic.

Multimedia Appendix 14 Growth stunting awareness video created with the DG Murray Trust, South Africa, 2018.

## Abbreviations

CHW: community health worker
CME: continuing medical education
E-E: entertainment-education
ELM: Elaboration Likelihood Model
HCD: human-centered design
LMIC: low- and middle-income countries
RTHB: Road to Health Book
